# Inhibition of triple negative breast cancer-associated inflammation, tumor growth and brain colonization by targeting monoacylglycerol lipase

**DOI:** 10.1038/s41598-022-09358-8

**Published:** 2022-03-29

**Authors:** Othman Benchama, Sergiy Tyukhtenko, Michael S. Malamas, Mark K. Williams, Alexandros Makriyannis, Hava Karsenty Avraham

**Affiliations:** 1 The Center for Drug Discovery, Northeastern University, 360 Huntington Avenue, Boston, MA 02115 USA; 2 Mineralogic LLC, Davis Square, Somerville, MA USA

**Keywords:** Breast cancer, Metastasis, Cancer, Drug discovery

## Abstract

While the prevalence of breast cancer metastasis in the brain is significantly higher in triple negative breast cancers (TNBCs), there is a lack of novel and/or improved therapies for these patients. Monoacylglycerol lipase (MAGL) is a hydrolase involved in lipid metabolism that catalyzes the degradation of 2-arachidonoylglycerol (2-AG) linked to generation of pro- and anti-inflammatory molecules. Here, we targeted MAGL in TNBCs, using a potent carbamate-based inhibitor AM9928 (hMAGL IC_50_ = 9 nM) with prolonged pharmacodynamic effects (46 h of target residence time). AM9928 blocked TNBC cell adhesion and transmigration across human brain microvascular endothelial cells (HBMECs) in 3D co-cultures. In addition, AM9928 inhibited the secretion of IL-6, IL-8, and VEGF-A from TNBC cells. TNBC-derived exosomes activated HBMECs resulting in secretion of elevated levels of IL-8 and VEGF, which were inhibited by AM9928. Using in vivo studies of syngeneic GFP-4T1-BrM5 mammary tumor cells, AM9928 inhibited tumor growth in the mammary fat pads and attenuated blood brain barrier (BBB) permeability changes, resulting in reduced TNBC colonization in brain. Together, these results support the potential clinical application of MAGL inhibitors as novel treatments for TNBC.

## Introduction

Breast cancer is a common cause of brain metastases occurring in at least 10–16% of patients and reaching 25–35% of TNBC patients^1–5^. Unfortunately, patients who are diagnosed with brain metastases often have poor prognosis with short overall survival times^6^. Triple negative breast cancer (TNBC) more frequently affects younger patients and have higher prevalence in African-American and Hispanic women^6,7^. TNBC tumors are larger in size and more biologically aggressive with lymph node involvement. TNBC patients often have a higher rate of distant recurrence and a poorer prognosis than patients with other breast cancer subtypes^8^.

Breast cancer metastases in brain (BCM/B) show significant morphological and genomic heterogeneity^1–6,9–13^. Patients with BCM/B have high mortality resulted from the brain lesions and are resistant to chemotherapy treatments^9,10^. Metastatic breast tumor cells transmigrate across the blood–brain barrier (BBB) and form colonies of tumor cells in brain^4,9,10^. Under normal conditions, the BBB is a highly selective barrier due to existence of tight junctions (TJs) between adjacent brain microvascular endothelial cells (BMECs). However, in the process of invasion of tumor cells to the brain, inflammatory cytokines and chemokines secreted by these infiltrating tumor cells disrupt the BBB integrity. Although the BBB prevents the delivery of most therapeutic drugs into the brain, the circulating metastatic tumor cells invade the damaged BBB to form colonies in the brain^9,10^.

Several genes were shown to be involved in the development of brain metastases which include cyclooxygenase COX-2, EGFR ligand HBEGF and α-2,6-sialyltransferase ST6GALNAC5^9,14^, all involved in facilitating cancer cell passage through the BBB. We have previously reported the roles of the proinflammatory peptide P in impairing the BBB integrity and the angiogenic factor Angiopoietin-2 in mediating activation of brain microvascular endothelial cells and BBB impairment, resulting in infiltration of TNBCs into the brain^15,16^. Further, loss of E-cadherin was found in breast cancer metastasis and its expression inversely correlates with tumor stage, pathologic stage, and prognosis of cancers of epithelial origin^17^.

The tumor microenvironment (TME) is important in cancer progression^10–12^. TME is characterized by chronic inflammation which stimulates tumorigenesis, especially in inflammatory breast cancer, where metastasis arises at the initial stage. TME is comprised of cells surrounding the tumor (such as macrophages, fibroblasts, and endothelial cells) and the extracellular matrix (ECM). TME in tumor niches differs from the healthy tissue microenvironments in cell type composition and phenotype^10–12^. TME promotes tumor development by complex signaling molecules that include soluble secreted molecules such as cytokines, chemokines, growth factors, proinflammatory enzymes, exosomes and matrix remodeling proteinases^10–13,18^. Interestingly, extracellular vesicles, a heterogeneous group of cell-derived membranous structures comprising of exosomes and microvesicles, were shown recently to breach intact BBB via Transcytosis^19^. Although tumor-derived exosomes are being recognized as essential mediators of intercellular communication between cancer and immune cells, it is not known whether exosomes derived from breast cancer cells can activate directly brain endothelial cells.

Cancer cells endogenously synthesize 95% of free fatty acids (FFAs) de novo^4^ and incorporate and remodel exogenous palmitate into structural and oncogenic glycerophospholipids, sphingolipids and ether lipids^4^. Fatty acid synthesis (FAS) pathway confers a survival advantage to cancer cells and especially to tumor cells that are resistant to chemotherapy^4^. Fatty acid-binding protein 5 (FABP5) promoted lipolysis droplets, de-novo fatty acid synthesis, and coordinated lipid signaling that promoted prostate cancer metastasis^20^. Increased FFA levels in tumors lead to enhanced tumor aggressiveness by increasing lipid synthesis mediated by lipolytic pathways^4,21–23^. Thus, targeting lipid metabolism may prevent the gain of survival advantage and improve treatment response in cancer cells.

MAGL is a serine hydrolase that regulates a fatty acid network that promotes cancer pathogenesis by enriching pro-tumorigenic signaling molecules. MAGL is a key hydrolytic enzyme in the FFA tumor network reported in colorectal cancer, neuroblastoma, nasopharyngeal carcinoma, and other cancers^19,21–23^. MAGL primarily hydrolyzes endocannabinoid 2-AG and other monoacylglycerides, and accounts for about 85% of 2-AG degradation, while the remaining part is degraded by α/β-hydrolase domain containing (ABHD) 6/12 enzymes. In addition, MAGL indirectly controls the levels of free fatty acids derived by their hydrolysis and other lipids derived by metabolism of fatty acids with pro-inflammatory or pro-tumorigenic effects^23^. Recently, 2-AG was shown to be the source for arachidonic acid, the precursor of prostaglandins and other inflammatory mediators^24–26^. Accumulated studies showed that MAGL inhibition impaired cell migration, invasion and tumorigenicity in some types of cancers^20–23^. Further, MAGL promoted progression of hepatocellular carcinoma via NF-kB mediated epithelial-mesenchymal transition^27^. Taken together, although MAGL was shown to be involved in some cancers and inflammatory diseases, its specific roles in TNBC tumor progression and the brain metastasis process is still unknown.

A new series of highly potent carbamate MAGL inhibitors were synthesized and characterized at the Center for Drug Discovery, Northeastern University. In this study, we have investigated the carbamate-based inhibitor AM9928, which exhibited high potency against recombinant human MAGL (hMAGL) with IC_50_ value of 8.9 nM. AM9928 contains a carbamate pharmacophore which binds covalently to Ser122. Upon binding of inhibitor to the MAGL active site, Ser122 attacks the carbonyl carbon of the carbamate group, triggering the formation of a covalent adduct. This results in a stable enzyme-ligand adduct rendering the enzyme incapable of further hydrolytic effects. The structure and mechanism of AM9928 has been published in the PhD thesis of Dr. Nasr (see link http://hdl.handle.net/2047/D20238396). Together, AM9928 demonstrated: (1) potency for the target; (2) suitable physicochemical properties with low lipophilicity (ClogP 3–4); (3) good microsomal (> 20 min) and plasma stability (> 120 min); and (4) potentially prolonged pharmacodynamic effects as determined by ^1^H NMR spectroscopy, assessing the time required for MAGL reactivation (residence time) following the covalent interaction between ligand and protein.

Since MAGL is important for lipid metabolism which plays various roles in tumorigenesis, we investigated MAGL’s role in TNBC growth and tumor colonization in brain. MAGL is highly expressed in TNBCs, which secrete high levels of inflammatory cytokines and chemokines. We hypothesized that tumor growth in the mammary fat pads and tumor cell infiltration across the BBB is facilitated via inflammatory chemokines/cytokines secreted from TNBC cells, leading to activation of HBMECs and resulting in TNBC spreading and colonization in brain. Here, we have performed studies using several human and mouse cell lines: (1) a TNBC human cell line (MDA-MB-231 cells); (2) human and mouse brain-seeking TNBC cell lines (MDA-MB-BrM2 and GFP-4T1-BrM5, respectively; (3) human brain microvascular endothelial cells (HBMECs); and (4) the spontaneous breast metastasis mouse models (syngeneic). We found that TNBC adhesion to HBMECs and transmigration across HBMECs was inhibited by AM9928. AM9928 inhibited TNBC’s secretion of inflammatory cytokines such as IL-6 and IL-8, and the angiogenic factor VEGF-A. Notably, AM9928 inhibited i*n vivo* changes in BBB permeability and decreased TNBC colonization in brain. Taken together, these results demonstrate novel mechanisms by which MAGL mediates its effects on brain metastasis by activation of brain microvascular endothelium and modulating BBB permeability. These studies support the potential of clinical application of MAGL inhibitors as a novel treatment of TNBC tumor growth and TNBC-colonization in the brain.

## Results

### Assessment of the residence time of AM4301 and AM9928

**Figure 1:**
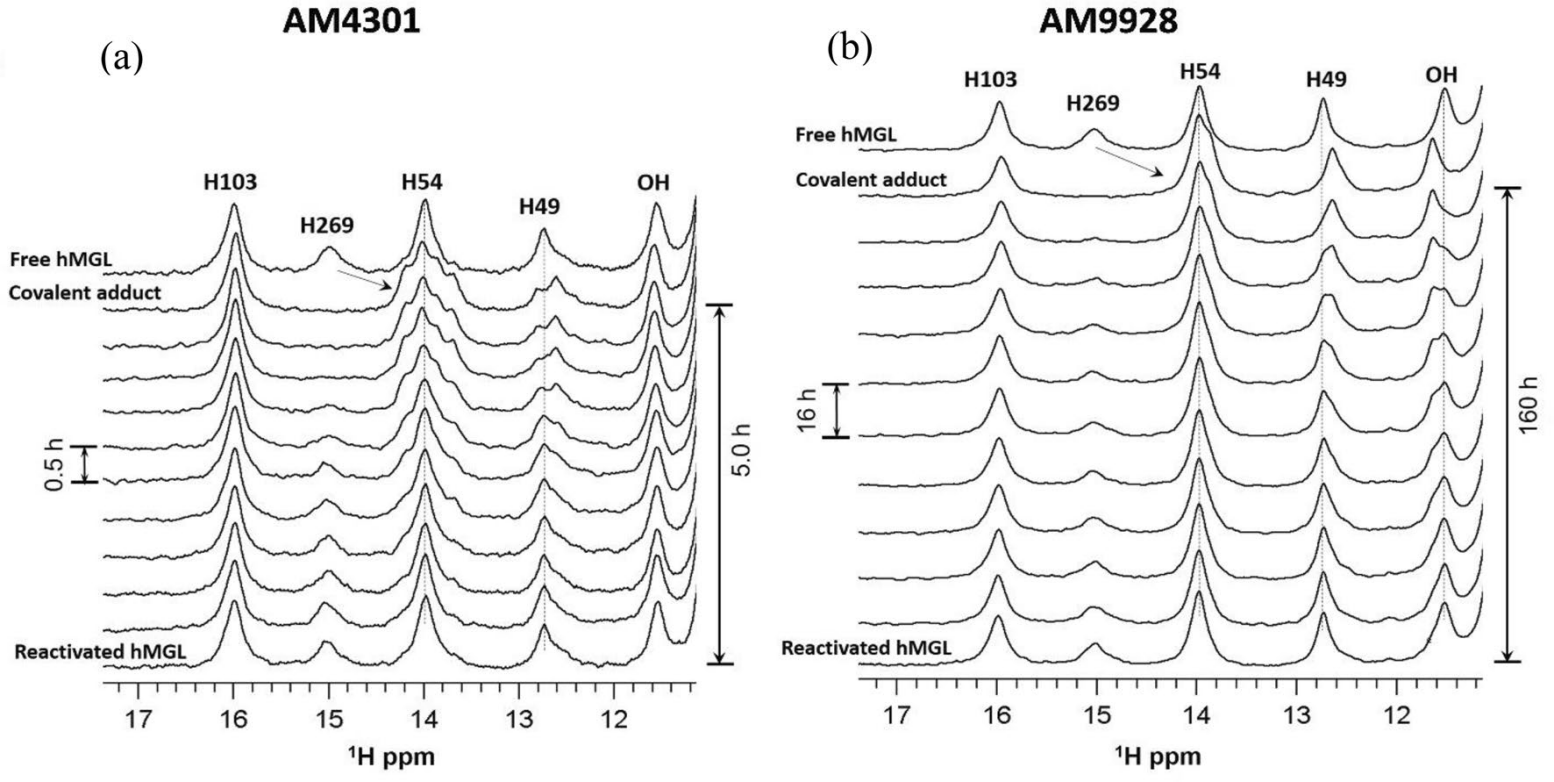
Downfield ^1^H NMR spectra of hMAGL. Formation of covalent adducts with the MAGL inhibitors and complete reactivation of the enzyme due to their hydrolysis: (**a**) AM4301; (**b**) AM9928.

In vitro binding and unbinding of potent hMAGL carbamate-based inhibitor AM9928, as compared to another MAGL inhibitor AM4301, was monitored by ^1^H NMR spectroscopy using downfield ^1^H NMR spectral pattern as a probe of conformational changes associated with binding^28–30^. The chemical shift perturbations (CSPs) induced by binding in the characteristic downfield NMR pattern are shown in Fig. 1. Addition of equimolar amounts of inhibitor in both cases caused significant upfield chemical shift (from 14.9 to ~ 13.9 ppm) for the resonance peak corresponding to the catalytic His269 and smaller shifts for other downfield resonances. Following the fast formation of hMAGL covalent adducts with AM4301 (Fig. 1a) and AM9928 (Fig. 1b), slow reactivation of the enzyme due to hydrolytic decomposition of adducts was monitored by real-time NMR spectroscopy. The residence time (lifetime) of ligand on a target is equal to the lifetime of covalent adduct which is a period required for adduct to decrease to 1/e (0.367) of its original concentration. The estimated residence time based on NMR time course data for AM4301 is 1.4 h whereas AM9928 residence time is 46 h. Significantly longer in vitro residence time of AM9928 in comparison with AM4301 could be translated to its prolonged pharmacodynamic effect. Based on these observations we selected AM9928 for our studies.

### The toxicity effects and time kinetics of treatment with AM9928

**Figure 2:**
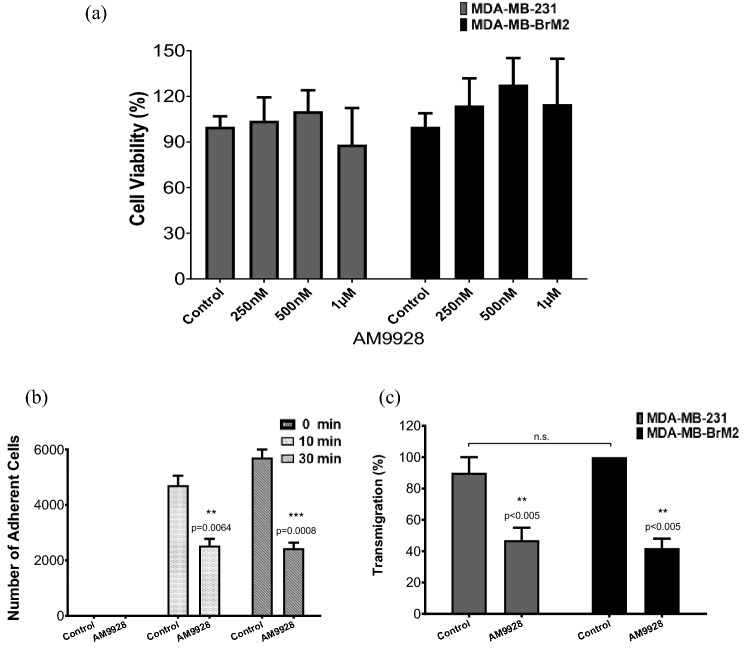
Effects of AM9928 on cell survival, adhesion, and trans-endothelial migration of TNBC cells across HBMEC cultures. (**a**) Effect of AM9928 on MDA-MB-231 and MDA-MB-BrM2 Cell Viability. AM9928 at different concentrations as noted (250 nM, 500 nM, or 1 μΜ) were incubated with 5 × 10^4^ cells/well for 72 h and treated as detailed in the manufacturer’s protocol (Abcam Cat# ab211091, MTT assay) to determine cell viability. Absorbance was measured at OD 590 nm using Biotek reader. Error bars represents the mean ± S.D. (**b**) Adhesion: After maintaining HBMECs at confluence for 5 days, approximately 40,000 labeled MDA-MB-BrM2 cells were seeded over the monolayer in the presence or absence of AM9928 and were allowed to adhere to HBMEC monolayer for either 0, 10 min or 30 min, as indicated. The TGL (a triple-modality reporter gene of thymidine kinase (T), GFP (G), and luciferase (L)TGL-tagged cells were then counted and compared to control (untreated) cells. Data shown are representative of at least three independent assays performed on duplicate or triplicate wells. The error bars indicate standard deviations. (**c**) Transmigration: After maintaining HBMECs at confluence for 5 days, approximately 40,000 labeled MDA-MB-231 or MDA-MB-BrM2 cells were seeded over the monolayer in the presence or absence of AM9928 for 5 h. Green labeled, transmigrated cells across the HBMEC were then counted and expressed as the percentage of cells transmigrated relative to control. Data shown are representative of at least three independent assays performed on triplicate wells. The error bars indicate standard deviations. *n.s* not significant.

First, the toxicity of AM9928 on TNBC cells were analyzed. TNBC cells include the highly invasive MDA-MB-231 and the brain- seeking variant MDA-MB-BrM2 cells. The following doses were examined: 250, 500 or 1000 nM, for durations of 72 h. No significant toxicity of AM9928 (Fig. 2a) was observed on the tested cells.

### AM998 inhibited adhesion and transmigration of breast cancer cells through human brain microvascular endothelial cells (HBMECs)

Next, in vitro adhesion assay was performed using co-cultures of the brain-seeking variant MDA-MB-BrM2 cells and HBMECs in the presence of vehicle control or AM9928. MDA-MB-BrM2 cells adhered to the untreated monolayer by as early as 10 min (Fig. 2b). Pre-treatment of HBMECs monolayers with AM9928 (100 ng/mL) blocked the adhesion of the tumor cells at time points of 10 and 30 min (Fig. 2b). We then examined the role of MAGL in transmigration of TNBC cells across HBMECs using an in vitro model where HBMECs were co-cultured with MDA-MB-231 cells or with MDA-MB-BrM2 cells^16^. Tumor cell transmigration across HBMECs was significantly inhibited (about 50%) by AM9928 (Fig. 2c).

### MAGL mediated effects on chemokines/cytokines

Cytokines are produced by both immune cells and cancer cells^31,32^. To characterize the potential activity of MAGL inhibitor AM9928 on the inflammatory responses in breast cancer, we used TNBC cells, the MDA-MB-231 and MDA-MB-BrM2 cells, as compared to the immortalized normal breast epithelial MCF-10A cells.

**Figure 3:**
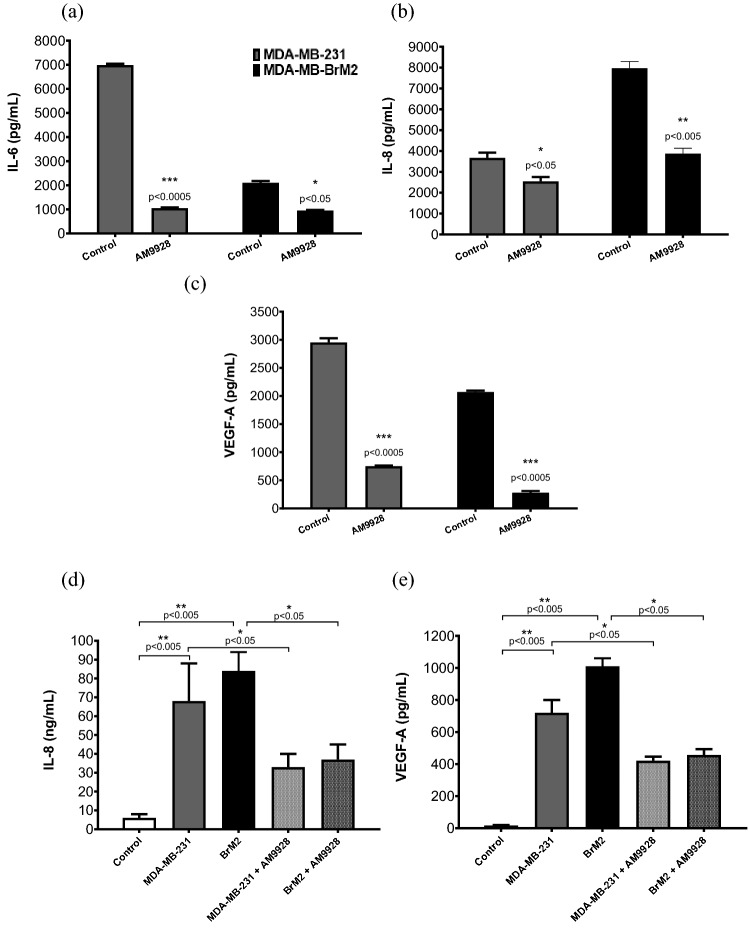
Expression levels of IL-6, IL-8, and VEGF-A in TNBC cells and in BMEC cells following exposure to exosomes derived from TNBC cells. (**a**)–(**c**): Expression levels of IL-6, IL-8, and VEGF-A in MDA-MB-231 and MDA-MB-BrM2 cells: cell supernatant samples were prepared from breast cancer cells treated with vehicle control or with AM9928, as indicated and the levels of the human cytokines/chemokines were measured using specific ELISA assays for human IL-6, IL-8, and VEGF-A (R&D Systems ^®^ELISAs). Data shown are representative of at least three independent assays performed on triplicate wells. The error bars indicate standard deviations. *p < 0.05 as compared to vehicle control, **p < 0.005 as compared to vehicle control and ***p < 0.0005 as compared to vehicle control. (**d**)–(**e**): Expression levels of IL-8 and VEGF-A in HBMECs: Exosomes were isolated from either MDA-MB-231 or MDA-MB-231BrM2 cells treated with vehicle control or with AM9928 for 24 h as indicated. Exosomes were then isolated and were incubated with human BMECs in 3D-cocultures for 6 h. Supernatants from HBMECs were collected after 24 h and analyzed for IL-8 and VEGF-A using ELISA assays. Data shown are representative of at least three independent assays performed on triplicate wells. The error bars indicate standard deviations. *p < 0.05 in untreated exosomes derived from MDA-MB-231 cells or MDA-MB-231-BrM2 cells as compared to TNBC cells treated with AM9928 and **p < 0.005 as compared to HBMECs control, as indicated in the figure.

First, we examined the effects of AM9928 as compared to the vehicle control on secretion of cytokines/chemokines in cells treated with either the vehicle control or with AM9928 (250 nM) for 72 h. The supernatants were collected and analyzed by specific ELISA assays for IL-6, IL-4, INF-alpha2, TGF-alpha, TNF-alpha, and IL-8. We observed significant inhibitory effects of AM9928 on IL-6 and IL-8 (Fig. 3a, b). Both inflammatory cytokines are important in tumor-associated inflammation^4,32–35^.

Second, we analyzed the effects of AM9928 on the angiogenic factor VEGF-A secretion and observed significant inhibitory effects of AM9928 on VEGF-A secretion (Fig. 3c).

Third, to determine if chemokines/cytokines are directly involved in activation of HBMECs, without the contact of tumor cells, we prepared TNBC-derived exosomes untreated or treated with AM9928 and tested their direct effects on HBMECs. MDA-MB-231 and MDA-MB-BrM2 derived exosomes caused activation of HBMECs leading to secretion of elevated levels of the pro-inflammatory IL-8 and VEGF-A. AM9928 treated TNBC-derived exosomes significantly inhibited activation of HBMECs, as observed by the secretion levels of IL-8 and VEGF-A (Fig. 3d, e).

### In vivo analysis of tumor growth in mammary fat pads and colonization of TNBCs in brain

**Figure 4:**
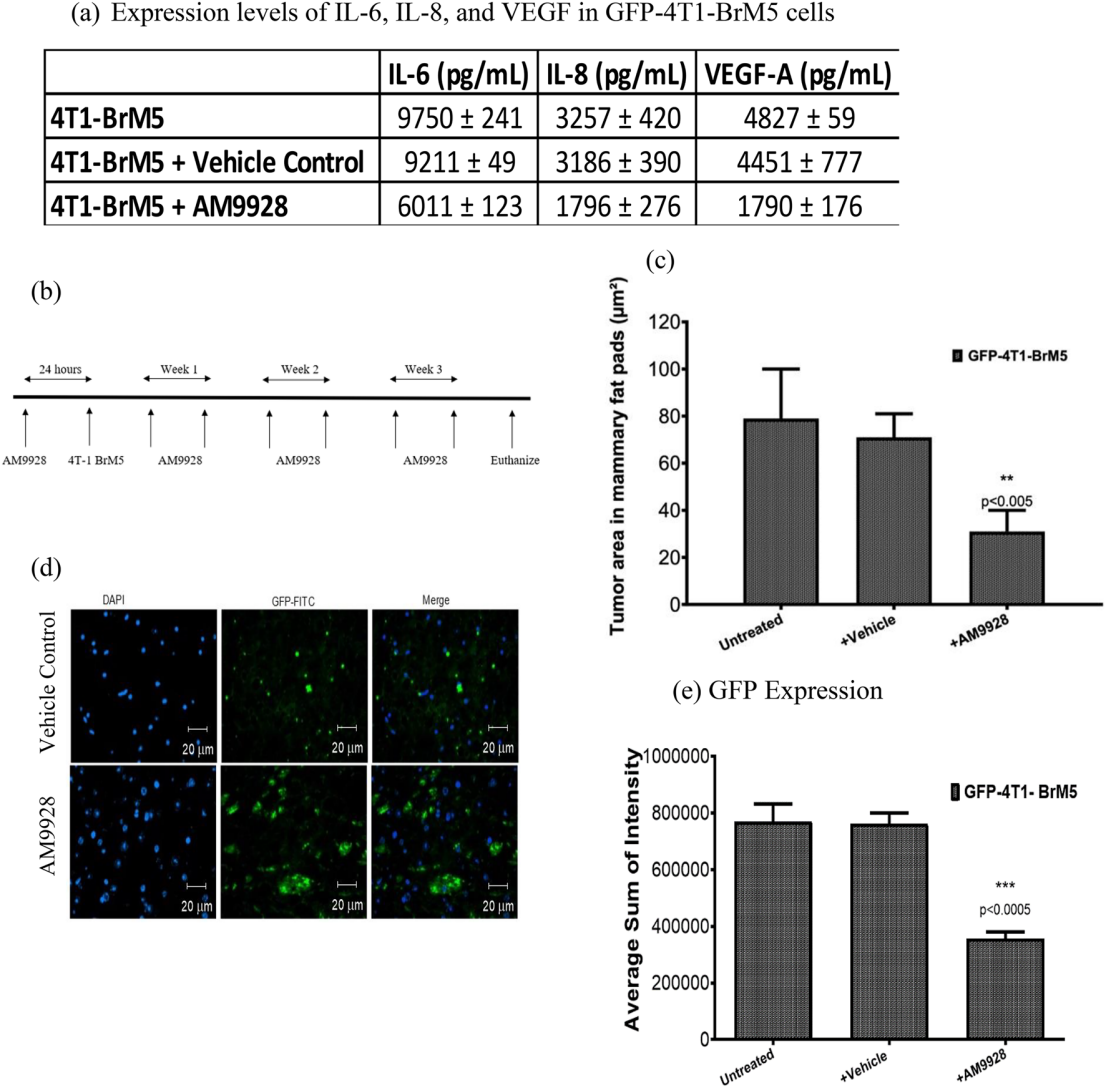
In vivo effects of AM9928 on TNBC tumor growth and brain metastasis: (**a**) Expression levels of IL-6, IL-8, and VEGF in GFP-4T1-BrM5 cells untreated and following treatment with AM9928: Cell supernatant samples were prepared from GFP-4T1-BrM5 cells either treated with vehicle control or with AM9928. The levels of the murine cytokines/chemokines were measured using specific ELISA assays for IL-6, IL-8, and VEGF-A (R&D Systems ^®^ELISAs). Data shown are representative of at least three independent assays performed on triplicate wells. The error bars indicate standard deviations. *p < 0.05 as compared to vehicle control, **p < 0.005 as compared to vehicle control and ***p < 0.0005 as compared to vehicle control. (**b**) Schematic presentation of time injection of AM9928 and GFP-4T1-BrM5 cells: AM9928 (at 10 mg/kg) or vehicle control were injected by I.V. twice a week for 3 weeks. (**c**) Tumor formation in mammary fat pads following treatment with AM9928: Murine tumor formation in mammary fat pads were measured at day 28: *n* = 10 mice/treatment; *p < 0.05, Mann–Whitney U‐test; **p < 0.005 as compared to vehicle control. (**d**) Detection of GFP-positive cells in brain tumors: GFP–4T1-BrM5 tumor cells were administered to the mammary fat pads as compared to control mice and were treated with vehicle control or with AM9928. Murine tumor cells in brain were detected by GFP immunostaining (GFP antibodies 1:50 dilutions; Abcam)(green) and their respective controls were used under the same standardized conditions. Brain nuclei were counterstained with DAPI (blue). *n* = 10 mice/treatment; These are representative images of over 50 images from three independent experiments. Scale bar = 20 μm. (**e**) Quantitative analysis of tumor cells in the brain: Murine tumor cells in brain were detected by GFP immunostaining as described above. The tumor areas at day 28 were detected by immunostaining with GFP antibody: *n* = 10 mice/treatment; *p < 0.05, Mann–Whitney U‐test; **p < 0.005 as compared to vehicle control.

For these studies, we employed the spontaneous breast cancer metastasis mouse model (syngeneic) of mammary tumor cells GFP-4T1-BrM5 cells, which “home” to brain and form breast metastasis in brain in mice. The GFP-4T1-BrM5 cells secreted IL-6, IL-8, and VEGF, which were inhibited by AM9928 (Fig. 4a). These results were like those obtained with MDA-MB-BrM2 cells (Fig. 3). In addition, AM9928 had similar effects on GFP-4T1-BrM5 cell survival, adhesion, and transmigration, as those observed with MDA-MB-BrM2 cells (data not shown). Therefore, we employed GFP-4T1-BrM5 cells for the in vivo studies.

To examine the effects of AM9928 on TNBC tumor growth and BBB integrity, GFP-4T1-BrM5 cells were administered (5 × 10^4^ cells) into the mammary fat pads of BALB/c mice and were injected with either the vehicle control or with AM9928 as shown in Fig. 4b (*n* = 10 mice per group per treatment). The GFP-4T1-BrM5 tumor cell growth in mammary fat pads and tumor cell seeding in the brain was observed by in vivo imaging and immunohistochemistry. Tumor growth in mammary fat pads was significantly reduced in mice treated with AM9928 as compared to mice treated with vehicle control (Fig. 4c). Quantitation of GFP–4T1‐BrM5 tumor cell colonization in brain was determined by ex vivo imaging of the brain and by immunohistochemistry using GFP antibody. The expression of GFP positive tumor cells was significantly lower in the mice group treated with AM9928, while the presence of GFP–4T1‐BrM5 tumor cells in brain was significantly higher in the untreated GFP-4T1-BrM5 cells treated mice or the vehicle control treated mice (Fig. 4d, e). Of note, we did not observe any effects of AM9928 on body weight of mice or toxicity in the treated AM9928 mice, as compared to untreated mice or mice treated with vehicle control (data not shown).

**Figure 5:**
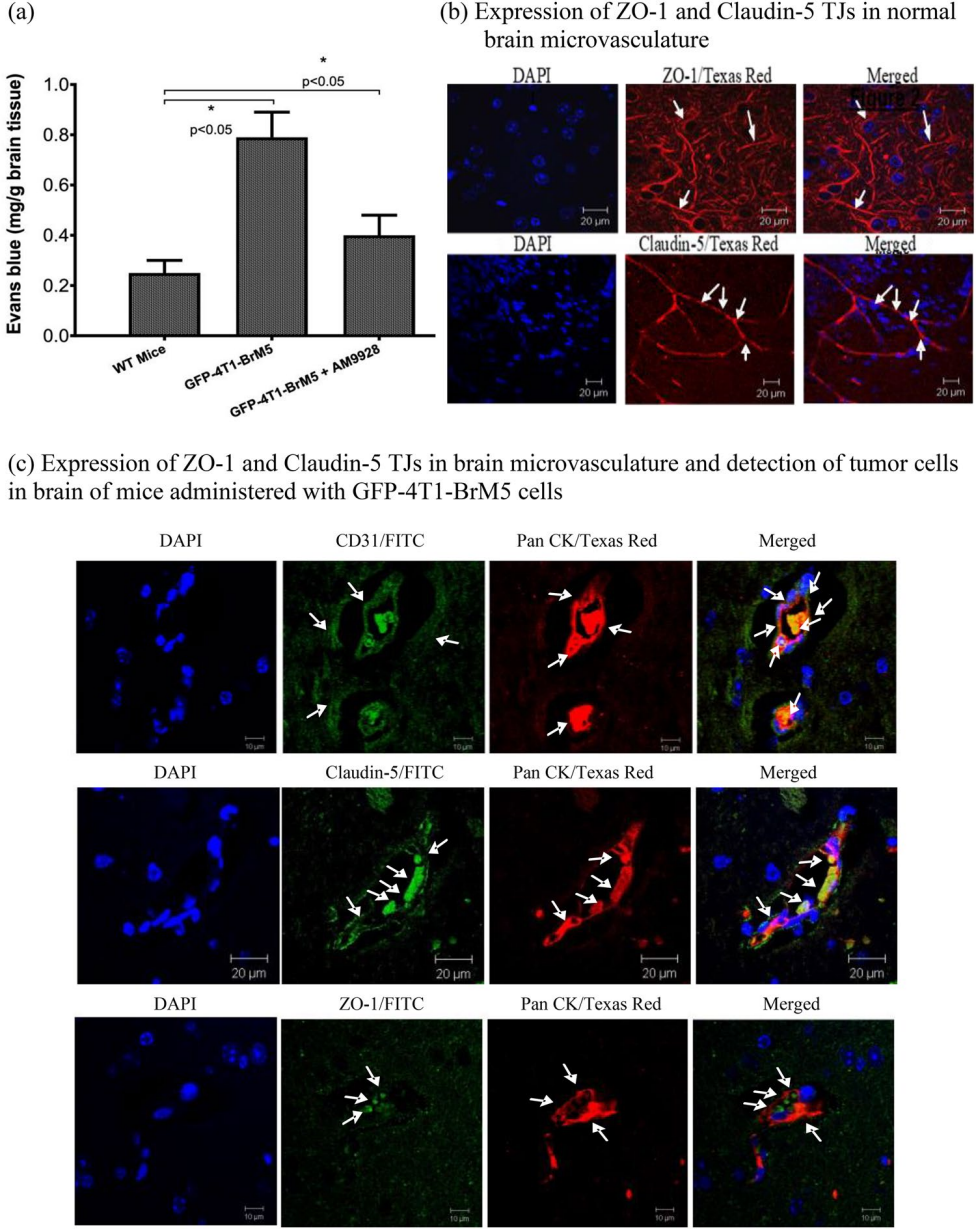
In vivo effect of AM9928 on transmigration of GFP-4T1-BrM5 cells across the BBB and on BMEC-Tight Junctions expression. (**a**) In vivo analysis of the BBB permeability: following injection of Evans blue, a statistically significant increase of the dye was found in the hippocampal region in mice administered with GFP-4T1-BrM5 cells and treated with vehicle control, but not in mice treated with AM9928. The level of dye in the control WT mice is shown also for comparison between the groups. This is a representative experiment of three separate experiments. The error bars indicate standard deviations. *p < 0.05 as compared to WT mice control. (**b**) Immunodetection of tight junctions in normal brain CD31 positive microvasculature (BMECs). The normal brain microvasculature was immunoassayed with CD31 antibody (1:500 dilution) as a marker for BMECs, using FITC- conjugated secondary antibody. Brain sections were immunoassayed for ZO-1(1:500 dilution) and Claudin-5 (1:500 dilution) using fluorescence Texas Red secondary antibody to detect ZO-1 and Claudin-5 expression in normal mouse brain sections. Arrows show the intact ZO-1(red) and Claudin-5 (red) structures, as indicated. Brain nuclei were counterstained with DAPI (blue). These are representative images of over 50 fields examined in three independent experiments. Scale bar = 20 μm. (**c**) In vivo effects of GFP-4T1-BrM5 tumor cells on the BBB integrity and on tight junctions’ structures: upper panel: the tumor cells were detected by pan-cytokeratin (Pan-CK) antibody (1:500 dilution) as primary antibody and fluorescence Texas red was used as a secondary antibody to detect the tumor cells in brain sections of mice administered with GFP-4T1-BrM5 cells. The brain microvasculature was immunoassayed with CD31 antibody (1:500 dilution) to detect BMECs (red). The arrows indicate the tumor cells or the CD31 BMECs as indicated. These are representative images of over 50 fields examined in three independent experiments. Magnification—× 40; scale bar-10 μm. Middle and Lower Panels: Immunodetection of tight junctions in brain tumors: Expression of Claudin-5 (middle panel) (green) and ZO-1 (lower panel) (green) in tumor brain sections were performed by immunostaining using FITC-conjugated secondary antibody as indicated. Detection of GFP-4T1-BrM5 cells in vivo was performed by immunostaining with Pan-CK antibody (red). Co-immunostaining of the tumor cells with Claudin-5 or with ZO-1 are shown in the merged figures. Brain nuclei were counterstained with DAPI (blue). These are representative images of over 50 fields examined from three independent experiments. The magnification—× 40; scale bar-10 μm.

Increased in BBB permeability was found in mice administered with tumor cells or with vehicle treated mice, while mice treated with AM9928 had lower BBB permeability changes as compared to control mice at day 28 (Fig. 5a). We then analyzed BMEC-tight junction proteins’ expression of ZO-1 and Claudin-5 in vivo in normal brain, as compared to TJs in tumor brains from syngeneic GFP-4T1-BrM5 mammary tumor cell model. ZO-1 is the main scaffolding protein and Claudin-5 is known as the TJ sealer protein of the BBB. As shown in Fig. 5b, both expression of ZO-1 and Claudin-5 were abundant in the normal brain vasculature. Following GFP–4T1‐BrM5 cell administration to the mammary fat pads, the GFP-tagged tumor cells migrated across the BBB after about 4 weeks. The GFP tagged tumor cells were detected by immunostaining with either Monoclonal Pan-Cytokeratin (Pan-CK) antibodies or GFP antibodies. No staining of normal brain sections with Pan-ck antibodies was observed (data not shown). These tumor cells were usually located around the brain capillaries and in-close proximity to BMECs (BMECS were detected with CD31 antibodies) (Fig. 5c).

**Figure 6:**
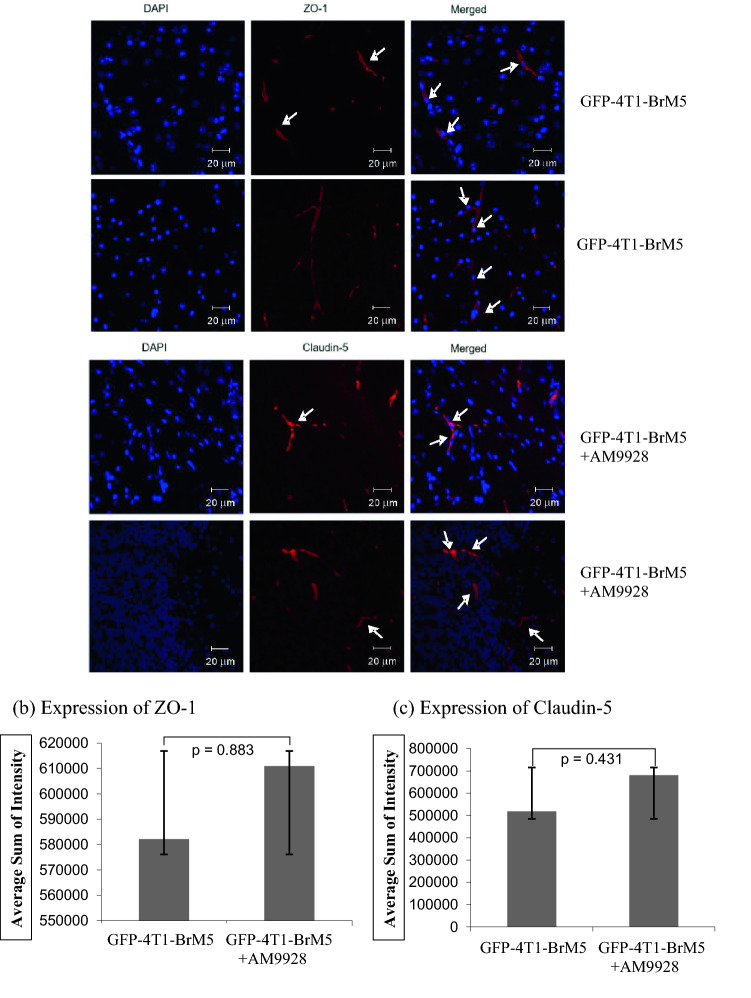
(**a**) Analysis of ZO-1 and Claudin-5 expression in the BBB in mice treated with AM9928: Fluorescence Texas Red was used as a secondary antibody to detect ZO-1 and Claudin-5 expression in mouse brain tumor sections. ZO-1 and Claudin-5 structures (red) are shown by arrows, as indicated. Brain nuclei were counterstained with DAPI (blue). These are representative images of over 50 fields examined from three independent experiments. The magnification—× 40; scale bar-20 μm. (**b**), (**c**): Quantitative analysis of Claudin-5 and ZO-1 proteins expression in the brain in mice treated with AM9928: The expression level was quantitated and compared with GFP-4T1-BrM5 injected mice and mice treated with vehicle control. Changes in BMEC-TJ proteins in brain section samples after AM9928 treatment as compared to BMEC-TJ proteins from control mice is shown. Data are presented as the mean ± S.D. of two experiments. Number of mice per group per treatment, n = 10. Scale bar = 20 μm.

Both ZO-1 and Claudin-5 structures were damaged in the brain tumor vasculature (Fig. 6a) as compared to the control brain (Fig. 5b). In some images, both ZO-1 and Claudin-5 structures withing BMECs were seen to colocalized with tumor cells, indicating the proximity of BMECs to the tumor cells in vivo. Interestingly, following treatment with AM9928, the expression of both ZO-1 and Claudin-5 was less impaired as compared to mice treated with vehicle control (Fig. 6a). The number of CD31^+^ vessels expressing TJ proteins, ZO-1 and Claudin-5, was performed and images were analyzed using Adobe Photoshop CS2. The expression of ZO-1, as determined by average sum of intensity was 582,096 and following treatment with AM9928 the average sum of intensity was 610,920 (Fig. 6b). The average sum of intensity of Claudin-5 expression in untreated group was 518,599 and following treatment with AM9928 the average sum of intensity was 681,457 (Fig. 6c). Although these differences in TJ numbers were not statistically significant between the untreated mice and the treated AM9928 mice, both ZO-1 and Claudin-5 were observed as less damaged structures in the AM9928 treated mice, as compared to the vehicle treated mice.

In summary, the number of mice positive with mammary tumors and brain tumors was significantly higher in the vehicle treated group as compared to AM9928 treated mice (Table 1). Importantly, the number of alive mice at day 28 was higher in the AM9928 treated mice as compared to the vehicle control group (Table 1). Thus, AM9928 significantly inhibited changes in the BBB permeability and reduced TNBC colonization in brain.

**Table 1:** In vivo effects of AM9928 on survival of mice following TNBC administration: summary of number of mice either treated with vehicle control or with AM9928 on days 22 and 28 as indicated.

Group	Number of mice with:		
	Mammary tumors (Day 22)	Brain metastasis (Day 28)	Survived mice (Day 28)
Vehicle treated	20	16	3
AM9928 treated	12	8	7

The number of mice with mammary fat tumors and brain tumors are shown.

The number of mice that survive at day 28 are indicated.

## Discussion

During tumor progression, cells undergo reprogramming of metabolic pathways that regulate glycolysis and the production of lipids^23,36–38^. The energy supply of lipids comes from de novo synthesis rather than circulating lipids during tumor development, which are modulated and regulated by MAGL^36–40^. Since MAGL is important in assigning the lipid stores in the direction of pro-tumorigenic signaling lipids in cancer cells^39,40^, we studied the mechanisms by which MAGL contributes to promoting TNBC metastases.

MAGL plays a major role in the endocannabinoid metabolism in addition to its lipolytic functions. MAGL provides a large pool of arachidonic acid (AA), from which pro-inflammatory prostaglandins are generated^41^. 2-AG, CB1/CB2 receptors and MAGL are key regulators of the endocannabinoid system involve in regulation of various metabolic pathways in vivo. By inhibiting MAGL, 2-AG levels are increased in tumor cells and in tumor niches, leading to changes in immune cell behavior in the TME and resulting in altered immune cell profile within the tumor niches being shifted from pro-inflammatory profile to a more anti-tumorigenic profile^41^. The effects of 2-AG on immune cells and TME usually involve CB2 cannabinoid receptors, although CB1 independent effects such as leukotriene biosynthesis and LTB4 activation could be observed^41^. MAGL is important for CB1 receptor (de)sensitization and the endocannabinoid tone.

Assessment of MAGL inhibitors for their in vitro potency was performed to select compounds for the in vitro and in vivo studies. The application of protein-based real-time ^1^H NMR spectroscopy allowed us to determine the residence time on target for potent MAGL inhibitor AM9928 (~ 46 h) (Fig. 1). The longer inhibitory effect of AM9928 strongly suggest prolonged pharmacodynamic effect of AM9928 which is required for our in vivo studies. Although previous studies^42^ showed that AM9928 lacked affinity for the cannabinoid receptors CB1 and CB2 receptors, it is important to exclude any effect of AM9928 on CB1 and/or CB2 receptors. Therefore, future studies will test the effects of AM9928 on mice lacking CB1/CB2 receptors.

Breast cancer progression and its responses to therapy are regulated by inflammation that promotes or suppresses tumor progression. While chronic inflammation initiates tumor progression and facilitates treatment resistance, acute inflammation stimulates the maturation of dendritic cells and antigen presentation, leading to increased anti-tumor immune responses. Acute and chronic inflammation are regulated by interferons, the interleukins, the chemokine family, mesenchymal growth factors, the tumor necrosis factor family and adipokines^43–45^. Key pro-inflammatory cytokines involved in inflammation include interleukin-1 (IL-1), IL-6, tumor necrosis factor (TNFα), IL-8, IL-12, IFN-γ and IL-18^43,46–50^. Therefore, we analyzed the effects of MAGL on these inflammatory cytokines/chemokines. Based on our results, AM9928 inhibited the secretion of tumor-inflammatory molecules IL-6 and IL-8 and the angiogenic factor VEGF-A in MDA-MB-231 cells and MDA-MB-BrM2 cells (Fig. 3) as well as the murine GFP-4T1-BrM5 cells (Fig. 4a). The tumor niches are also the source of proinflammatory cytokines^4,10^, which are secreted by both autocrine and paracrine manner though activation of STAT3^45^. The strong inhibition of IL-6 by AM9928 indicates the potential of AM9928 to target inflammation associated with TNBC. Therefore, MAGL may regulate lipid quality and/or quantity to promote aggressiveness such as migration and inflammation in breast cancer cells.

VEGF-A/VEGF-R signaling functions as an important survival pathway in breast cancer cells^51–53^. VEGF-A promoted tumor cell self-renewal through VEGF-A/VEGF-R/STAT3 signaling resulting in activation of Myc, Sox2 and STAT3^51–53^. High VEGF-A levels strongly correlated with both STAT3 and Myc expression as well as with tumor metastatic potential. Interestingly, we found significant inhibition of VEGF-A expression by AM9928 in human and murine TNBC cells (Figs. 3c, 4a), strongly suggesting the effectiveness of targeting MAGL in inhibition of angiogenesis.

Breast cancer-derived exosomes accumulate in the lung, spleen, and bone marrow of naïve mice. At these sites, the exosomes content rich in IL-6 causes pro-metastatic alterations associated with reduction of T cell proliferation, NK cell cytotoxicity and modulation of immune responses. However, the role of TNBC-derived exosomes on HBMECs is not known. Here we show that HBMECs were activated by TNBC-derived exosomes which resulted in secretion of IL-8 and VEGF-A, thereby facilitating tumor transmigration across HBMEC, and promoting tumor colonization. Further, AM9929 treated TNBC-derived exosomes inhibited HBMECs activation as indicated by the secretion levels of IL-8 and VEGF-A. Thus, TNBC-derived exosomes may act as important mediators of intercellular communication between TNBCs and brain endothelium facilitating the formation of pre-metastatic niches and contributing to tumor dissemination in brain and tumor angiogenesis.

Since MAGL is expressed in TNBCs and in the immune and stromal cells of the tumor microenvironment, inhibition of MAGL in both compartments is essential for inhibition of tumor growth and pathogenesis. Indeed, recent studies showed that MAGL deficiency in the tumor microenvironment slows tumor growth in non-small cell lung cancer^41^. Further, the MAGL inhibitor JZL184 was shown to reduce osteolytic bone metastasis in mouse models of advanced breast and prostate cancer and osteosarcoma, and inhibited skeletal tumor growth, metastasis, and the formation of ectopic bone in models of osteosarcoma^54^. Inhibition of breast cancer MDA-231-BT cell motility in vitro by JZL184 (JZL, 10 μM) and MAGL knockdown prolonged the survival in mice injected with metastatic osteosarcoma cancer cells^54^. These results are similar to those obtained in our model system of TNBC, showing that AM9928 targeted both the tumor cells and the tumor niches and inhibited tumor growth in the mammary fat pads (Fig. 4). Future studies in our lab will examine if AM9928 has similar effects as JZL 184 in inhibition of cancer-related bone damage.

The permeability of the BBB plays a crucial role in brain metastasis and is implicated in the initial stages of cancer cell extravasation. In the presence of invading tumor cells, the local BBB is altered and exhibits heterogeneous permeability^14–16^. The changes in permeability of the BBB‐BMEC‐TJs are a dynamic process dependent on the interaction between TNBCs and BMECs. Our results showed that TNBC transmigration across the BBB resulted in increased BBB permeability (Fig. 5). These effects could be mediated by IL-6 and IL-8 as well as VEGF, which were secreted by tumor cells and were shown to influence the BBB permeability^15,16^. AM9928 mediated its effects on both the tumor cells and the tumor microenvironment including BMECs. Since AM9928 inhibited tumor growth in the mammary fat pads (Fig. 4) and prevented changes in BBB integrity in vivo*,* resulting in reduced tumor cell colonization in brain, we conclude that MAGL plays a role in TNBC tumor growth and TNBC transmigration across the BBB, in addition to its roles in neurological and neurodegenerative diseases^25,26^. Since metastasis to the brain was reported to be associated with damage to the cranial bone (osteolysis)^1–3^, it is important to investigate whether AM9928 may have bone protective effects in mice bearing the GFP-4T1-BrM5 cells. We plan to examine this question in future studies in our lab.

Taken together, we suggest that targeting MAGL may provide a new approach to reducing expression of proinflammatory factors as well as inhibiting tumor growth and tumor cell colonization in brain.

## Methods

### Ethics statement

*The animal study protocol was approved by The Institutional Animal Care and Use Committee of BIDMC Institutional Animal Care and Use Committee (IACUC), protocol number 086-2011, in accordance with the Public Health Service (PHS) Policy on Humane Care and Use of Laboratory Animals and the* Guide for the Care and Use of Laboratory Animals (NIH), for the protection of Vertebrates Used for Scientific Purposes (Scientific Procedures) Act 1986 as well as the ARRIVE guidelines*. All animal studies were performed in BIDMC.*

### Breast cancer cell lines

Normal immortalized breast epithelial cells (MCF-10A) and the human breast cancer cell lines MDA-MB-231 were purchased from American Type Culture Collection (ATCC, Manassas, VA). MDA-MB-231 cells are TNBC cells lacking PR, ER and HER-2. The breast tumor metastatic brain variants include the MDA-231-BrM2 tagged to GFP as well as the murine mammary tumor cells GFP- 4T1-BrM5 which specifically form metastatic brain tumors^14–16^ and generate multifocal lesions in the cerebrum, the cerebellum, and the brainstem, with features typical of brain metastasis in cancer patients. All cell lines were incubated in 5% CO_2_ at 37 °C, as described (16).

### MAGL inhibitor AM9928

MAGL inhibitor AM9928 was synthesized and characterized at the Center for Drug Discovery, Northeastern University. This compound was tested with both species of human and rat MAGL to examine the inhibitory effects. Further, this compound was tested against human FAAH and human ABHD6^42,55^. To this end, AM9928 inhibited human MAGL (hMAGL) with an IC_50_ value of 8.9 nM. AM9928 covalently inactivates MAGL by carbamylation of the enzyme's catalytic Ser122 nucleophile. The structure and mechanism of action of AM9928 has been published in the PhD thesis of Dr. Nasr (see link http://hdl.handle.net/2047/D20238396).

### Nuclear magnetic resonance spectroscopy and data analysis

Target-based 1D ^1^H-NMR spectroscopy was employed for real-time monitoring of the unbinding kinetics. NMR experiments were performed at physiological conditions (37 °C, pH7.4) on Bruker AVANCE 700 MHz spectrometer equipped with a 5-mm triple resonance inverse probe. For the efficient detection of exchangeable downfield resonances, a standard 3–9-19 WATERGATE pulse sequence (p3919pgp) with gradients and additional flipback pulse was used. This sequence significantly reduces unwanted attenuation of peak intensities originated from spin diffusion and chemical exchange with water. Samples of highly purified recombinant hMAGL prepared for NMR binding/unbinding experiments (0.5–0.6 mL) were 0.2–0.3 mM in 95% H_2_O, 5% D_2_O buffer containing 20 mM sodium phosphate, 100 mM NaCl and 5 mM TCEP (tris(2-carboxyethyl)phosphine) at pH7.4. Ligand titration into the target enzyme was performed using 50 mM stock solutions of inhibitors dissolved in DMSO-d6. Chemical shift referencing was done by using internal DSS (sodium 2,2-dimethyl-2-silapentane-5-sulfonate) standard (20 μM). The data were visualized and processed using Bruker software Topspin 3.2. For all NMR spectra an exponential multiplication (broadening factor lb = 30 Hz) was applied. Characteristic CSPs in the downfield region of hMAGL proton NMR spectra were observed because of fast and complete covalent adduct formation when the potent inhibitor is titrated into the protein in a 1:1 ratio. During subsequent hydrolytic ligand unbinding the integral intensity of the merged peak at ~ 14.0 ppm, which is equal to two protons (His269 and His54), gradually decreases to the value of one proton corresponding to the state when the enzyme is fully reactivated. Integration of this peak allows reliable measurements of decreases in adduct concentration over a period. The NMR peak at 15.9 ppm (His103) was chosen as a reference for integration procedure since its position is not affected upon adduct formation. Plots of the natural logarithm of the adduct concentration versus time yielded a linear relationship for examined inhibitors, indicating pseudo first-order kinetic behaviors (data not shown). In this case, the slope of curve represents the enzyme reactivation constant (k_r_) which allows evaluation of the half-life (t_1/2_ = ln2/k_r_) of covalent adducts as well as residence time (τ = 1/k_r_) of ligands on the target enzyme.

### Isolation of exosomes

The culture supernatants of MDA-MB-231 and MDA-MB-BrM2 cells untreated or treated with AM9928 for 24 h, at approximately 65% confluence and were harvested after 16 h conditioning in serum-free media. Cells and debris were cleared from the supernatants by centrifugation (500 g, 10 min) followed by filtration using 0.22-micron filters (Millipore Inc.). Exosomes were prepared from cell-free supernatants using the Exosome Isolation Kit (Kit# EIK-01, Creative Biolabs, NY). The quantitative and qualitative analysis of exosomes were performed on double sandwich enzyme-linked immunoassay using the Total Exosome Capture & Quantification Kit (Kit#EQK-04Creative Biolabs).

### In vivo effects of MAGL on BBB integrity and TNBC colonization in brain

Female (6 weeks) BALB/c mice were purchased from Jackson Laboratories (Bar Harbor, ME). The mice were housed at an AAALAC-accredited facility at Beth Israel Deaconess Medical Center, Boston. The mice were handled in accordance with the animal care policy of Harvard Medical School. Mice were euthanized humanely by CO_2_ inhalation in the end of the experiments following treatment and tumor samples were harvested for further study as described below.

We have selected AM9928 for our in vivo studies based on its prolonged target engagement. We expected that the prolonged inhibitory effect of AM9928 would be translatable to a longer pharmacodynamic effect when compared to other MAGL inhibitors such as AM4301.To that end, we studied the effects of AM9928 on BMEC-TJs and tumor colonization in the brain using the spontaneous breast cancer metastasis mouse model (syngeneic) of mammary tumor cells. We used GFP-4T1-BrM5 cells which migrate to the brain^14–16^ and form breast metastasis in the brains of Balb/c mice. We administered GFP-4T1-BrM5 tumor cells (5 × 10^4^ cells) into the mammary fat pads of Balb/c mice. More than 70% of these mice developed mammary tumors within 3 weeks, while brain microtumors were developed in about 5 weeks. Here, following administration of GFP-4T1-BrM5 cells, mice were injected with AM9928 (10 mg/kg, i.v.) or the vehicle control twice a week for 3 weeks (10 mice/group/treatment). Tumor sizes in the mammary fat pads were measured using calipers, and the volume was calculated, using the formula: V = 0.52 × (length) × (width)^2^. The BBB integrity at the end of the experiments was analyzed by Evan blue test in control groups and in mice treated with AM9928 as compared to vehicle control. Briefly, Evans’s blue (EB) (Sigma Chemical Co. St. Louis, MO. USA.) dye (25% in 0.9% NaCl solution) was intravenously injected at dose of 25 mg/kg under anesthesia. One hour after the injection, animals were sacrificed. Brains were weighed, clipped, and individually placed within formamide p.a. (2 mL/brain). The content of dye extracted from each brain was determined by spectrophotometer (Photometer 4010, Boehringer) at 620 nm and compared to standard graph created through the recording of optical densities from serial dilutions of EB in 0.9% NaCl solution. For in vivo imaging, spectral fluorescence images were obtained using a Maestro‐based imaging system (CRI Inc., Woburn, MA, USA). Five sets of side‐by‐side whole‐brain images of animals with tumors from the GFP-4T1-BrM5 cells treated with vehicle control or with AM9928 were obtained. In the end of the experiment, mice were sacrificed, and brain and mammary fat pad tissues were collected for further analysis.

### Statistical analysis

All values are expressed as the mean ± SEM of the mean and are representative of two to three independent experiments as indicated. *P* values were calculated using unpaired two-tailed Student’s tests, built in the GraphPad Prism Software for evaluation of statistical significance.

### Ethical approval

All in vivo studies were performed in the Division of Experimental Medicine, BIDMC, Harvard Medical School, Boston and all in vitro studies were performed in CDD at Northeastern University. All in vivo studies were approved by BIDMC institutional Animal care and use committee (IACUC) and the mice were handled in accordance with the animal care policy of BIDMC, Harvard Medical School and in accordance with the Declaration of Helsinki.

## Data Availability

All data generated or analyzed during this study are included in this published article.

## Abbreviations

AA: Arachidonic acid
AEA: Anandamide
ABHD6 and ABHD12: α/β-hydrolase domain containing (ABHD) 6/12 enzymes
BBB: Blood–brain barrier
BC: Breast cancer
BCM/Brain: Breast cancer metastasis in brain
BCM: Breast cancer metastasis
BMEC: Brain microvascular endothelial cells
CB1: Cannabinoid receptor type 1
CB2: Cannabinoid receptor type 2
ECM: Extracellular matrix
ECS: Endocannabinoid system
ER: Estrogen receptor negative
FAS: Fatty acid synthesis
FFAs: Free fatty acids
HBMECs: Human brain microvascular endothelial cells
hMAGL: Human monoacylglycerol lipase
IF: Immunostaining
IHC: Immunohistochemistry
LCM: Laser capture microdissection
MAGL: Monoacylglycerol lipase
RT-PCR: Reverse transcription polymerase chain reaction
TEER: Trans-endothelial electrical resistance
TGL: A triple-modality reporter gene of thymidine kinase (T), GFP (G), and luciferase (L)
TJ: Tight junction
TME: Tumor microenvironment
TNBC: Triple negative and basal type breast cancer
VEGF-A: Vascular endothelial growth factor
WB: Western blotting

## References

[1] Hubalek M, Czech T, Muller H (2017). Biological subtypes of triple-negative breast cancer. Breast Care (Basel).

[2] Yam C, Mani SA, Moulder SL (2017). Targeting the molecular subtypes of triple negative breast cancer: Understanding the diversity to progress the field. Oncologist.

[3] Yates LR (2017). Genomic evolution of breast cancer metastasis and relapse. Cancer Cell.

[4] Omabe M, Ezeani M, Omabe KN (2015). Lipid metabolism and cancer progression: The missing target in metastatic cancer treatment. J. Appl. Biomed..

[5] da Silva JL, Cardoso Nunes NC, Izetti P, de Mesquita GG, de Melo AC (2020). Triple negative breast cancer: A thorough review of biomarkers. Crit. Rev. Oncol. Hematol..

[6] Jezequel P (2019). Identification of three subtypes of triple-negative breast cancer with potential therapeutic implications. Breast Cancer Res..

[7] Valiente M (2020). Brain metastasis cell lines panel: A public resource of organotropic cell lines. Cancer Res..

[8] Lehmann BD (2016). Refinement of triple-negative breast cancer molecular subtypes: Implications for neoadjuvant chemotherapy selection. PLoS ONE.

[9] Hu G, Kang Y, Wang XF (2009). From breast to the brain: Unraveling the puzzle of metastasis organotropism. J. Mol. Cell Biol..

[10] Cacho-Diaz B (2020). Tumor microenvironment differences between primary tumor and brain metastases. J. Transl. Med..

[11] Roma-Rodrigues C, Mendes R, Baptista PV, Fernandes AR (2019). Targeting tumor microenvironment for cancer therapy. Int. J. Mol. Sci..

[12] Belli C (2018). Targeting the microenvironment in solid tumors. Cancer Treat. Rev..

[13] Nakamura K, Smyth MJ (2017). Targeting cancer-related inflammation in the era of immunotherapy. Immunol. Cell Biol..

[14] Bos PD (2009). Genes that mediate breast cancer metastasis to the brain. Nature.

[15] Rodriguez PL, Jiang S, Fu Y, Avraham S, Avraham HK (2014). The proinflammatory peptide substance P promotes blood–brain barrier breaching by breast cancer cells through changes in microvascular endothelial cell tight junctions. Int. J. Cancer.

[16] Avraham HK (2014). Angiopoietin-2 mediates blood–brain barrier impairment and colonization of triple-negative breast cancer cells in brain. J. Pathol..

[17] Borcherding N (2018). Re-evaluating E-Cadherin and beta-Catenin: A pan-cancer proteomic approach with an emphasis on breast cancer. Am. J. Pathol..

[18] Cruceriu D, Baldasici O, Balacescu O, Berindan-Neagoe I (2020). The dual role of tumor necrosis factor-alpha (TNF-alpha) in breast cancer: Molecular insights and therapeutic approaches. Cell Oncol. (Dordr.).

[19] Morad G (2019). Tumor-derived extracellular vesicles breach the intact blood–brain barrier via transcytosis. ACS Nano.

[20] Carbonetti G (2019). FABP5 coordinates lipid signaling that promotes prostate cancer metastasis. Sci. Rep..

[21] Zhang H (2020). Monoacylglycerol Lipase knockdown inhibits cell proliferation and metastasis in lung adenocarcinoma. Front. Oncol..

[22] Li X (2019). Effect of monoacylglycerol lipase on the tumor growth in endometrial cancer. J. Obstet. Gynaecol. Res..

[23] Nomura DK (2010). Monoacylglycerol lipase regulates a fatty acid network that promotes cancer pathogenesis. Cell.

[24] Grabner GF, Zimmermann R, Schicho R, Taschler U (2017). Monoglyceride lipase as a drug target: At the crossroads of arachidonic acid metabolism and endocannabinoid signaling. Pharmacol. Ther..

[25] Zanfirescu A, Ungurianu A, Mihai DP, Radulescu D, Nitulescu GM (2021). Targeting monoacylglycerol lipase in pursuit of therapies for neurological and neurodegenerative diseases. Molecules.

[26] Deng H, Li W (2020). Monoacylglycerol lipase inhibitors: Modulators for lipid metabolism in cancer malignancy, neurological and metabolic disorders. Acta Pharm. Sin. B.

[27] Zhu W (2016). Monoacylglycerol lipase promotes progression of hepatocellular carcinoma via NF-kappaB-mediated epithelial-mesenchymal transition. J. Hematol. Oncol..

[28] Tyukhtenko S (2020). Conformational gating, dynamics and allostery in human monoacylglycerol lipase. Sci. Rep..

[29] Tyukhtenko S (2016). Specific inter-residue interactions as determinants of human monoacylglycerol lipase catalytic competency: A role for global conformational changes. J. Biol. Chem..

[30] Tyukhtenko S (2018). Effects of distal mutations on the structure, dynamics and catalysis of human monoacylglycerol lipase. Sci. Rep..

[31] Nagarsheth N, Wicha MS, Zou W (2017). Chemokines in the cancer microenvironment and their relevance in cancer immunotherapy. Nat. Rev. Immunol..

[32] King J, Mir H, Singh S (2017). Association of cytokines and chemokines in pathogenesis of breast cancer. Prog. Mol. Biol. Transl. Sci..

[33] Masjedi A (2018). The significant role of interleukin-6 and its signaling pathway in the immunopathogenesis and treatment of breast cancer. Biomed. Pharmacother..

[34] Banerjee K, Resat H (2016). Constitutive activation of STAT3 in breast cancer cells: A review. Int. J. Cancer.

[35] Atretkhany KN, Drutskaya MS, Nedospasov SA, Grivennikov SI, Kuprash DV (2016). Chemokines, cytokines and exosomes help tumors to shape inflammatory microenvironment. Pharmacol. Ther..

[36] Beloribi-Djefaflia S, Vasseur S, Guillaumond F (2016). Lipid metabolic reprogramming in cancer cells. Oncogenesis.

[37] Taib B (2019). Lipid accumulation and oxidation in glioblastoma multiforme. Sci. Rep..

[38] Yecies JL, Manning BD (2010). Chewing the fat on tumor cell metabolism. Cell.

[39] Guzman M (2010). A new age for MAGL. Chem. Biol..

[40] Tuo W (2017). Therapeutic potential of fatty acid amide hydrolase, monoacylglycerol lipase, and N-acylethanolamine acid amidase inhibitors. J. Med. Chem..

[41] Kienzl M (2021). Monoacylglycerol lipase deficiency in the tumor microenvironment slows tumor growth in non-small cell lung cancer. Oncoimmunology.

[42] Alapafuja S (2019). Synthesis and evaluation of potent and selective MGL inhibitors as a glaucoma treatment. Bioorg. Med. Chem..

[43] Wang W, Nag SA, Zhang R (2015). Targeting the NFkappaB signaling pathways for breast cancer prevention and therapy. Curr. Med. Chem..

[44] Karki R, Man SM, Kanneganti TD (2017). Inflammasomes and cancer. Cancer Immunol. Res..

[45] Bahiraee A, Ebrahimi R, Halabian R, Aghabozorgi AS, Amani J (2019). The role of inflammation and its related microRNAs in breast cancer: A narrative review. J. Cell. Physiol..

[46] Ma Y (2017). IL-6, IL-8 and TNF-alpha levels correlate with disease stage in breast cancer patients. Adv. Clin. Exp. Med..

[47] Mouchemore KA, Anderson RL, Hamilton JA (2018). Neutrophils, G-CSF and their contribution to breast cancer metastasis. FEBS J..

[48] Lim B, Woodward WA, Wang X, Reuben JM, Ueno NT (2018). Inflammatory breast cancer biology: The tumour microenvironment is key. Nat. Rev. Cancer.

[49] Taniguchi K, Karin M (2018). NF-kappaB, inflammation, immunity and cancer: Coming of age. Nat. Rev. Immunol..

[50] Sau A, Cabrita MA, Pratt MAC (2018). NF-kappaB at the crossroads of normal mammary gland biology and the pathogenesis and prevention of BRCA1-mutated breast cancer. Cancer Prev. Res. (Phila.).

[51] Huang P (2018). Chemotherapy-driven increases in the CDKN1A/PTN/PTPRZ1 axis promote chemoresistance by activating the NF-kappaB pathway in breast cancer cells. Cell Commun. Signal.

[52] Mery B (2019). Advocacy for a new oncology research paradigm: The model of Bevacizumab in triple-negative breast cancer in a French cohort study. Oncology.

[53] Banys-Paluchowski M (2018). The clinical relevance of serum vascular endothelial growth factor (VEGF) in correlation to circulating tumor cells and other serum biomarkers in patients with metastatic breast cancer. Breast Cancer Res. Treat..

[54] Marino S (2019). Paradoxical effects of JZL184, an inhibitor of monoacylglycerol lipase, on bone remodelling in healthy and cancer-bearing mice. EBioMedicine.

[55] Miller S (2016). Harnessing the endocannabinoid 2-arachidonoylglycerol to lower intraocular pressure in a murine model. Investig. Ophthalmol. Vis. Sci..

